# An *In Vivo* Zebrafish Model for Interrogating ROS-Mediated Pancreatic *β*-Cell Injury, Response, and Prevention

**DOI:** 10.1155/2018/1324739

**Published:** 2018-03-28

**Authors:** Abhishek A. Kulkarni, Abass M. Conteh, Cody A. Sorrell, Anjali Mirmira, Sarah A. Tersey, Raghavendra G. Mirmira, Amelia K. Linnemann, Ryan M. Anderson

**Affiliations:** ^1^Center for Diabetes and Metabolic Disease, Indiana University School of Medicine, Indianapolis, IN 46202, USA; ^2^Department of Biochemistry and Molecular Biology, Indiana University School of Medicine, Indianapolis, IN 46202, USA; ^3^Department of Pediatrics, Indiana University School of Medicine, Indianapolis, IN 46202, USA; ^4^Department of Cellular and Integrative Physiology, Indiana University School of Medicine, Indianapolis, IN 46202, USA

## Abstract

It is well known that a chronic state of elevated reactive oxygen species (ROS) in pancreatic *β*-cells impairs their ability to release insulin in response to elevated plasma glucose. Moreover, at its extreme, unmitigated ROS drives regulated cell death. This dysfunctional state of ROS buildup can result both from genetic predisposition and environmental factors such as obesity and overnutrition. Importantly, excessive ROS buildup may underlie metabolic pathologies such as type 2 diabetes mellitus. The ability to monitor ROS dynamics in *β*-cells in situ and to manipulate it via genetic, pharmacological, and environmental means would accelerate the development of novel therapeutics that could abate this pathology. Currently, there is a lack of models with these attributes that are available to the field. In this study, we use a zebrafish model to demonstrate that ROS can be generated in a *β*-cell-specific manner using a hybrid chemical genetic approach. Using a transgenic nitroreductase-expressing zebrafish line, *Tg(ins:Flag-NTR)^s950^*, treated with the prodrug metronidazole (MTZ), we found that ROS is rapidly and explicitly generated in *β*-cells. Furthermore, the level of ROS generated was proportional to the dosage of prodrug added to the system. At high doses of MTZ, caspase 3 was rapidly cleaved, *β*-cells underwent regulated cell death, and macrophages were recruited to the islet to phagocytose the debris. Based on our findings, we propose a model for the mechanism of NTR/MTZ action in transgenic eukaryotic cells and demonstrate the robust utility of this system to model ROS-related disease pathology.

## 1. Introduction

The generation of reactive oxygen species (ROS)—including peroxides, superoxides, and oxygen radicals—in excess of ROS mitigation mechanisms, results in cellular dysfunction and triggers regulated cell death under extreme circumstances [1]. Cells of metabolically active tissues are predisposed to high levels of ROS production, and thus, metabolic diseases such as type 2 diabetes mellitus (T2DM) are often associated with excessive ROS generation and resulting oxidative stress [2]. T2DM is characterized by chronic hyperglycemia resulting from the dysfunction of insulin-secreting pancreatic *β*-cells in the setting of overnutrition and obesity [3]. This dysfunction may be driven in part by the generation of excessive ROS, which likely results from the low endogenous levels of antioxidant enzymes in *β*-cells [4]. ROS diminishes the expression of insulin in *β*-cells, impairs glucose-stimulated insulin secretion, and promotes *β*-cell apoptosis [5, 6]. The availability of a vertebrate model to study factors that regulate ROS dynamics in the islet in situ would accelerate the discovery and testing of novel therapeutics for a variety of metabolic diseases, including T2DM. The zebrafish, *Danio rerio*, is a robust model to interrogate the pathogenesis of metabolic disease and the efficacy of experimental therapeutics [7, 8].

The oxygen-insensitive NAD(P)H nitroreductase (NTR, *NfsB*) enzyme, cloned from *E. coli*, has been harnessed to drive tissue-specific cell ablation in various transgenic zebrafish models. When NTR-expressing transgenic zebrafish lines, such as *Tg(ins:Flag-NTR)^s950^*, are treated with the antibiotic metronidazole (MTZ), this prodrug is metabolized into cytotoxins that are retained by NTR-expressing *β*-cells, rapidly inducing their death [9]. While the precise mechanisms of MTZ-induced cell toxicity have not been characterized in NTR-expressing transgenic lines, existing research provides some insight: nitroreduction of MTZ may produce cytotoxic nitroradical metabolites, which can crosslink DNA [10–12]. In this study, we use transgenic NTR^+^ zebrafish to demonstrate their suitability for modeling ROS generation, cellular responses to ROS, and pharmaceutical interventions *in vivo*. Together, our findings suggest that zebrafish are a superb model for ROS-related disorders.

## 2. Results

### 2.1. Metronidazole Induces *β*-Cell ROS Generation in an NTR- and Dose-Dependent Manner

**Figure 1:**
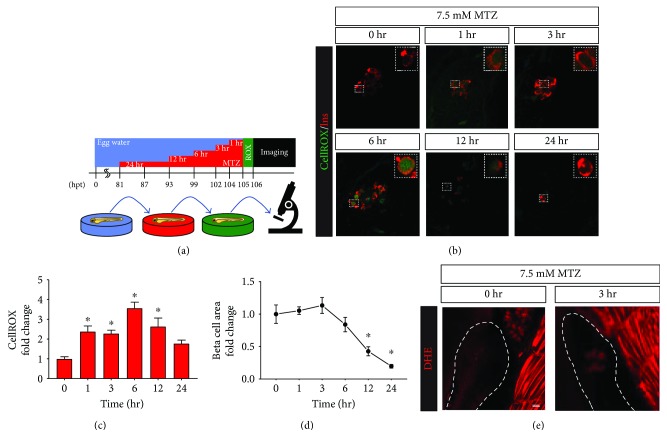
Time-dependent metronidazole induction of *β*-cell-specific ROS. (a) Schematic of MTZ treatments and imaging. Zebrafish (NTR^+^) larvae were treated with MTZ or vehicle for 0, 1, 3, 6, 12, or 24 hours with a “staggered start” such that all treatments were completed simultaneously; larvae were then incubated with CellROX green stain at 105 hpf and fixed/analyzed at 106 hpf. (b) Representative immunofluorescence images of zebrafish pancreatic islets stained with insulin antibody and CellROX green after 7.5 mM MTZ treatments. Magnified insets (bounded by dashed boxes) highlight the dose-dependent increase in CellROX green signal in *β*-cells. (c) Quantification of CellROX green intensity in insulin-positive *β*-cells showing a significant increase in ROS generation after 1, 3, 6, and 12 hours of MTZ treatment as compared to vehicle-treated controls (*n* = 12 for each condition). (d) MTZ treatment caused a significant decrease in *β*-cell area after 12 or 24 hours of treatment as compared to untreated controls. (e) Representative immunofluorescence images of zebrafish pancreatic islets treated for 3 hours with 0 or 7.5 mM MTZ and stained with 5 *μ*M DHE. Dotted lines demarcate the boundaries of the pancreas. Graphed data are presented as mean ± SEM (^∗^*p* < 0.05). Statistical significance was determined by one-way ANOVA followed by post hoc Holm-Sidak test. Scale bar indicates 10 *μ*m.

The nitroreductase-metronidazole (NTR-MTZ) system has been widely implemented as a tool to efficiently ablate cells in a tissue-specific and temporally controllable manner [13]. However, the molecular mechanisms driving its induction of regulated cell death are not fully understood. To determine if ROS are generated in MTZ-treated NTR-expressing cells, we used *Tg(ins:Flag-NTR)^s950^* transgenic zebrafish that express insulin promoter-driven NTR in the pancreatic *β*-cells. Heterozygous transgenic larvae were immersed in a solution of MTZ for 0, 1, 3, 6, 12, or 24 hr, then stained with CellROX green to indicate ROS (Figure 1(a)). Incubation start times were staggered such that all larvae were at 106 hours postfertilization (hpf) at analysis. We began treatments with 7.5 mM MTZ, a dose which is effective to ablate *β*-cells after 24 hr exposure [14]. With 1 hour of treatment, we observed ROS staining specifically in *β*-cell nuclei, whereas adjacent islet cells were not stained (Figure 1(b)). With longer treatments, ROS levels increased by almost 4-fold relative to untreated controls, reaching a peak intensity with 6 hours of treatment, then showing less increase with 12 or 24 hours of treatment (Figure 1(c)). We attribute this diminished staining to the attrition of *β*-cells via regulated cell death mechanisms and their clearance by phagocytes (Figure 1(d)), as well as the neogenesis of *β*-cells that have not yet generated detectable ROS. Importantly, neither MTZ nor the transgene was toxic alone; ROS generation in *β*-cells required both components ([9], data not shown). To further confirm that the CellROX green staining that we observed was truly representative of MTZ-induced cellular ROS and not artefactual (i.e., simply due to an interaction of CellROX green with the reduced nitroradical form of metronidazole), we next incubated MTZ-treated transgenic larvae in 5 *μ*M dihydroethidium (DHE). Upon its oxidation to 2OH-ethidium by superoxide, this cell-permeant dye is excited at 405 nm and emits a bright red fluorescence at 570 nm [15]. In 106 hpf *Tg(ins:Flag-NTR)^s950^* larvae that were not treated with MTZ, we detected no specific pancreatic fluorescence in any sample (Figure 1(e); *n* = 13). In contrast, in transgenic larvae treated with 7.5 mM MTZ for 3 hours, we observed strong fluorescence in *β*-cells in every case (Figure 1(e); *n* = 14). These data indicate that superoxide is generated in transgenic *β*-cells in response to MTZ.

**Figure 2:**
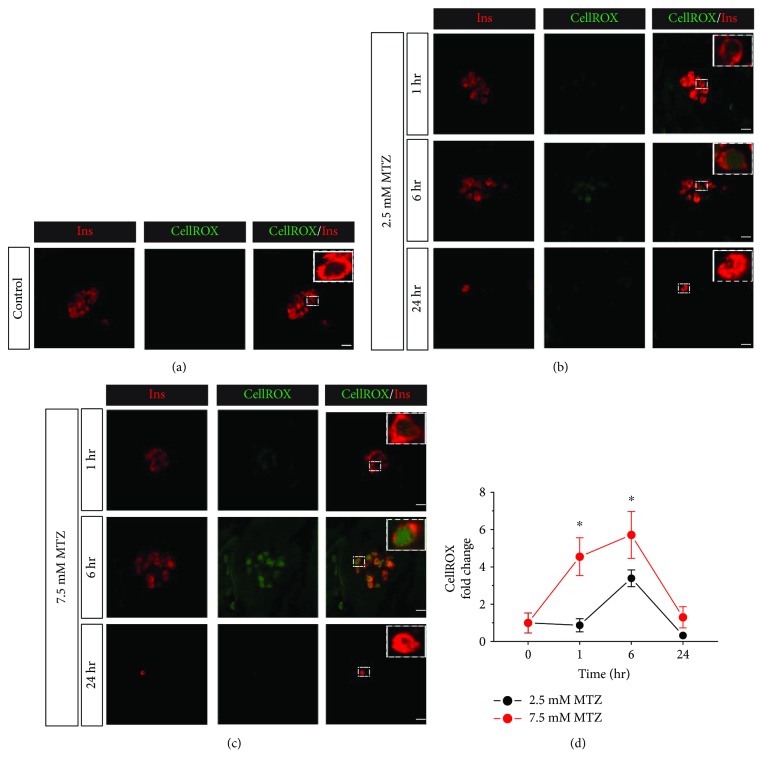
Metronidazole induces ROS generation in a dose-dependent manner. (a) Representative image of vehicle-treated zebrafish islets (*n* = 12) at 106 hpf. (b) Representative image of islets of zebrafish (NTR^+^) larvae (*n* = 12 per condition) treated with 2.5 mM and 7.5 mM MTZ at different time points. (c) Quantification of CellROX intensity shows a significant increase after 1 or 6 hours of treatment in the *β*-cells of 7.5 mM MTZ-treated embryos, as compared to untreated controls. Data are presented as mean ± SEM (^∗^*p* < 0.05). Statistical significance was determined by one-way ANOVA followed by post hoc Holm-Sidak test. Scale bar indicates 10 *μ*m.

Next, we hypothesized that the level of ROS generated in the NTR^+^* β*-cells would be directly dependent on the concentration of MTZ present. To investigate if there is a dose-dependent relationship, we treated embryos with both low (2.5 mM) and high (7.5 mM) concentrations of MTZ using the same experimental paradigm indicated in Figure 1(a). As expected, there was no ROS generation in the untreated control *β*-cells (Figure 2(a)). With 1 hour of treatment, the 2.5 mM dose did not show significant ROS generation, but 7.5 mM MTZ induced a more than 4-fold increase in ROS levels as compared to untreated controls (Figures 2(b)–2(d)). With 6 hours of treatment, the 2.5 mM treatment did not result in significant ROS generation, though it trended upward, while the 7.5 mM dose caused a nearly 6-fold rise in ROS levels relative to untreated controls. Consistent with our previous observations, the measured ROS levels were no different than baseline with 24-hour treatment of either the 2.5 mM or 7.5 mM MTZ. Despite the lower levels of ROS observed with 2.5 mM treatment, there was a dramatic reduction in the number of *β*-cells at the 24-hour time point (Figure 1(b)). Thus, even though induced ROS levels are lower with the 2.5 mM dose, this dose is sufficient to induce cell death over a 24-hour period.

### 2.2. MTZ-Induced ROS Generation Leads to Immune Cell Recruitment and *β*-Cell Apoptosis

**Figure 3:**
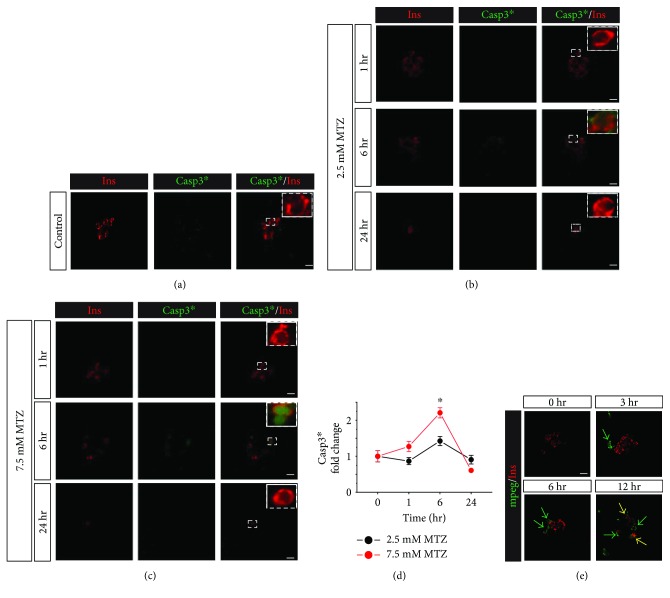
Metronidazole induces apoptosis signaling in *β*-cells. (a) Representative image of vehicle-treated zebrafish islets (*n* = 12) after fixing at 106 hpf. (b) Representative image of islets of zebrafish (NTR^+^) larvae (*n* = 12 per condition) treated with 2.5 mM or 7.5 mM MTZ at different time points and immune-stained for insulin and cleaved caspase 3 (Casp3^∗^). (c) Quantification of Casp3^∗^ intensity shows a significant increase after 6 hours of treatment in the *β*-cells of 7.5 mM MTZ-treated embryos compared to vehicle. (d) Representative immunofluorescence images of zebrafish (*mpeg+*) islets (*N* = 6/condition) treated with 7.5 mM MTZ showing macrophage invasion into islets (green arrows) and their engulfment of *β*-cells (yellow arrows). Data are presented as mean ± SEM (^∗^*p* < 0.05). Statistical significance was determined by one-way ANOVA followed by post hoc Holm-Sidak test. Scale bar indicates 10 *μ*m.

Many studies have correlated the production of ROS with the induction of apoptotic cell death [15–17]. Therefore, to determine whether the generation of ROS is correlated with the induction of *β*-cell apoptosis in this system, we analyzed cleaved caspase 3 (Casp3^∗^) in islet *β*-cells after following the same MTZ treatment paradigm shown in Figure 1(a). Casp3^∗^ is the active form of caspase 3 and an indicator of the activated apoptotic pathway. We did not detect significant Casp3^∗^ staining in the untreated controls or with a 1-hour MTZ treatment (Figures 3(a)–3(d)). However, with a 6-hour treatment, Casp3^∗^ was significantly increased with 7.5 mM MTZ, but not 2.5 mM relative to untreated controls, following a pattern similar to ROS generation (Figures 2(d) and 3(d)). As before, with 24 hours of treatment, almost all *β*-cells were ablated by both concentrations of MTZ (Figures 3(b) and 3(c)).

It has been demonstrated that ROS-injured *β*-cells release factors that attract immune cells [18]. Consistent with this finding, we show that generation of ROS in *β*-cells is coincident with the infiltration of *Tg(mpeg1:GFP) +* macrophages into the islet; this is first apparent with a 3-hour treatment, and peak infiltration is seen with 6 hours of MTZ treatment—when the ROS staining was also measured at its highest levels. Additionally, engulfment of *β*-cells by macrophages is first observed with a 12-hour MTZ treatment, a time point that is coincident with the observed drop in the *β*-cell area (Figures 1(d) and 3(e)).

### 2.3. Antioxidants Protect *β*-Cells from MTZ-Induced ROS Generation

**Figure 4:**
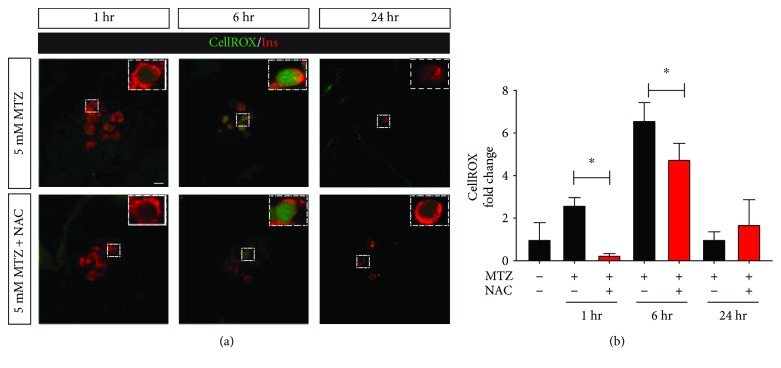
Antioxidant treatment protects from metronidazole-induced ROS generation in *β*-cells. Zebrafish larvae (*n* = 12 per condition) were treated with 5 mM metronidazole ± *N*-acetyl-L-cysteine (NAC) for 1, 6, or 24 hours followed by an assessment of ROS using CellROX green stain. (a) Representative images of islets of 106 hpf zebrafish (NTR^+^) embryos treated with 5 mM MTZ ± NAC at different time points. (b) Quantification of CellROX green intensity shows NAC-mediated protection from MTZ-induced ROS in *β*-cells after 1 or 6 hours of treatment. Data are presented as mean ± SEM (^∗^*p* < 0.05). Statistical significance was determined by Student's *t*-test. Scale bar indicates 10 *μ*m.

We hypothesized that the generation of ROS in *β*-cells could be mitigated in our zebrafish model by the addition of small molecule antioxidants to the water. To test this hypothesis, we used the common antioxidant *N*-acetyl-L-cysteine (NAC). We treated zebrafish larvae with an intermediate dose of 5 mM MTZ with the goal of using a dose that was strong enough to induce a rapid ROS response, but that would not overwhelm other treatments. MTZ treatments were supplemented with 100 *μ*M NAC and ROS intensity was measured at multiple time points (Figure 4). With either a 1- or 6-hour treatment of MTZ, NAC significantly reduced the levels of ROS staining in *β*-cells. Consistent with all other treatments, there was no significant difference with a 24-hour treatment, which again could be attributed to the ablation of nearly all *β*-cells in the presence of MTZ treatment alone. Together, we conclude that MTZ drives the production of ROS in *β*-cells in the presence of NTR. Additionally, a known antioxidant was effective at mitigating this effect, suggesting that other novel compounds might be uncovered through screening approaches in this zebrafish system.

## 3. Discussion

### 3.1. Mechanism of Cell Ablation by MTZ-NTR

**Figure 5:**
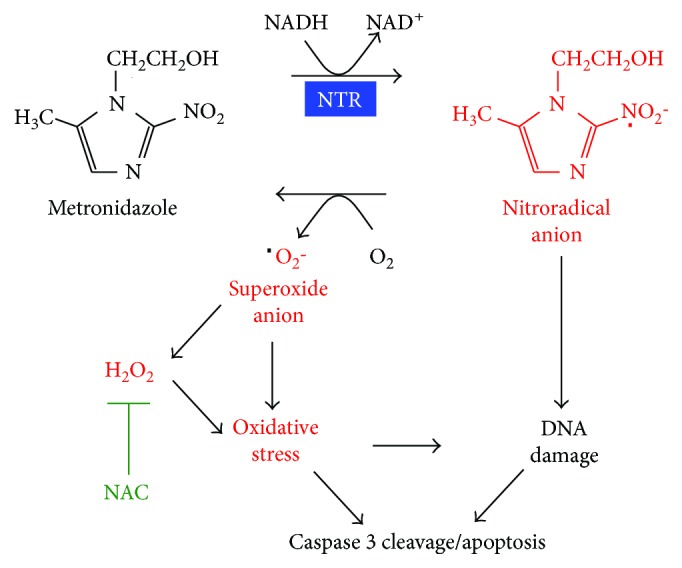
Proposed mechanism of metronidazole-nitroreductase-mediated cell ablation. In the aerobic setting of NTR-expressing eukaryotic cells, we propose that MTZ is reduced to a nitroradical anion by electron transfer from NADH, in a type 2-like mechanism. This radical may be cytotoxic and directly induces DNA damage and apoptosis. Alternately, this radical may regenerate back to metronidazole by electron transfer to O_2_, concurrently forming superoxide anion and ROS derivatives. This, in turn, drives increased cellular-oxidative stress and triggering of regulated cell death.

NTR (*NfsB*) is a type 1 oxygen-insensitive nitroreductase that catalyzes the full reduction of nitroaromatic compounds under anaerobic conditions [19]. In anaerobes, MTZ serves as a prodrug that is metabolized by NTR to generate cytotoxic derivatives capable of blocking DNA synthesis and inducing DNA damage [20]. In our study, we found that MTZ also induces ROS generation in the presence of NTR. This is consistent with the hypothesis that under aerobic conditions, as when expressed in mammalian cells, NTR might generate superoxide and derivative reactive oxygen species, potentially through a type 2-like “futile reduction cycle” (Figure 5) [21].

### 3.2. Utility of MTZ-NTR Zebrafish System as a Disease Model beyond Just Cell Ablation

As a *β*-cell ablation system, the relevance of Ins:NTR/MTZ to type 1 diabetes is evident. However, chronic ROS production and associated *β*-cell dysfunction are also critical to the pathology of type 2 diabetes even before *β*-cell mass is diminished. Intriguingly, because the generation of ROS by NTR in this model is dependent on the dose of MTZ treatment, this provides a compelling opportunity to manipulate ROS under varied contexts. For instance, many other disease conditions like type 2 diabetes, atherosclerosis, diabetic neuropathy, and cancer arise as a result of chronic ROS generation in specific tissues [22]. To model such cases, low concentrations of MTZ can be used for generating persistent ROS conditions and studying the effects. Future studies will determine whether lower levels of ROS can be induced by MTZ treatments that are sufficient to impair *β*-cell function, but not to induce cell death.

Zebrafish proves to be an outstanding model organism for studying ROS generation and ROS-related pathologies. The MTZ-NTR system seems to work exceptionally well for cell-specific ablation. However, with the added possibility of precisely modulating the ROS generation using MTZ dosing and antioxidants like NAC, this makes zebrafish a particularly flexible model.

## 4. Methods

### 4.1. Zebrafish Maintenance and Embryo Collection

Wild-type (AB), *Tg(ins:Flag-NTR)^s950^* (ZDB-ALT-130930-5) [23], and *Tg(mpeg1:GFP)* (ZDB-ALT-120117-1) zebrafish were maintained at 28.5°C in a recirculating aquaculture system enclosed in a cabinet and subjected to a 14-/10-hour light/dark cycle in accordance with institutional policies under IACUC oversight. Heterozygous outcrossed embryos bearing the *Tg(ins:Flag-NTR)^s950^* allele were collected at spawning and maintained in a 28.5°C incubator in egg water-filled petri dishes. Transgenic zebrafish larvae were genotyped by epifluorescence at 80 hpf using a Leica M205FA dissecting microscope.

### 4.2. Chemical Treatments

1-Phenyl-2-thiourea (PTU; Acros #207250250) supplementation at 0.003% was used to prevent pigmentation in all embryos after gastrulation stages. Metronidazole (Sigma #095K093) solutions of 2.5 mM, 5 mM, and 7.5 mM were prepared in egg water (0.1% instant ocean salt, 0.0075% calcium sulfate) that was supplemented with PTU. For antioxidant treatments, egg water, either with or without 5 mM MTZ, was supplemented with 100 *μ*M *N*-acetyl L-cysteine (NAC; Acros #160280250), which was diluted from a 100 mM stock prepared in ddH_2_O. Control treatments for MTZ and NAC used egg water alone.

### 4.3. ROS Staining, Immunofluorescence, and Image Collection

Cellular ROS was detected using a CellROX green reagent (Invitrogen #C10444); live larvae were transferred to 1.5 ml microcentrifuge tubes, washed with egg water, then incubated in the dark for 1 hr at 28.5°C with 10 *μ*M CellROX green diluted in egg water. At the conclusion of each experiment, larvae were washed in egg water then fixed with 3% formaldehyde in a PEM buffer (0.21 M PIPES, 1 mM MgSO_4_, 2 mM EGTA, and pH 7) at 4°C overnight. Fixed larvae were washed with PBS and deyolked, then antibody staining was performed as described [24]. The following concentrations of primary antibodies were used: 1 : 200 guinea pig anti-insulin (Invitrogen #180067), 1 : 200 rabbit anti-cleaved caspase 3 (Cell Signaling Technologies #9661S), and 1 : 100 mouse anti-glucagon (Sigma #SAB4200685). Primary antibodies were detected with 1 : 500 dilutions of complementary Alexa-conjugated secondary antibodies (Jackson ImmunoResearch). DNA was stained with TO-PRO3 (Thermo Fisher #T3605) diluted 1 : 500. After staining, larvae were mounted on slides in VECTASHIELD (Vector Labs H-1000), and confocal imaging was performed with a Zeiss LSM700 microscope. The confocal stacks of pancreatic islets were analyzed with CellProfiler software [25]. For detection of superoxide in situ in live pancreatic *β*-cells, heterozygous *Tg(ins:Flag-NTR)^s950^* larvae were treated with 7.5 mM MTZ for 3 hours then placed in 5 *μ*M dihydroethidium (Thermo Fisher #D1168) + 0.02% DMSO for 30 minutes in dark conditions. Larvae were then paralyzed with 0.01% tricaine (Sigma #A-5040), mounted on glass-bottom petri dishes (Mattek #P35G-0-10-C) in 0.5% low melt agarose (Sigma #A9414), and imaged with a Zeiss LSM700 confocal microscope.

### 4.4. Statistical Analysis

The data are presented as the means ± standard error of the mean (SEM). The data analyses were performed using the GraphPad Prism 7.1 software package. Significant differences between the mean values were determined using Student's *t*-test, where two means were compared, and one-way analysis of variance (ANOVA) followed by post hoc Holm-Sidak test when more than two means were compared. The differences were considered statistically significant at *p* < 0.05.
